# Impact of COVID-19-Related Personal Protective Equipment Changes on Dental Education: A Qualitative Study to Explore Faculty and Student Perspective

**DOI:** 10.1155/2024/5551126

**Published:** 2024-01-18

**Authors:** Shaista Rashid, Mohamed ElSalhy

**Affiliations:** ^1^Missouri School of Dentistry and Oral Health, A.T. Still University, St Louis, Missouri, USA; ^2^College of Dental Medicine, University of New England, Portland, Maine, USA

## Abstract

**Background:**

COVID-19 pandemic and its related personal protective equipment have impacted all aspects of dental education. The qualitative study assesses the impact of COVID-19-related changes and their effects on students' clinical learning from student and faculty perspectives.

**Methods:**

This qualitative study involved third- and fourth-year predoctoral dental students and full-time dental clinical faculty. A semistructured interview guide was used. The interview guide consisted of seven open-ended questions about the impact of the new COVID-19-related infection control procedures on students' learning experience in the dental clinic. Interviews were recorded, transcribed verbatim, and analyzed using a basic interpretative approach by two independent researchers. Emerged themes were identified.

**Results:**

Twelve faculty members and 21 students participated in six focus groups. Three major themes emerged from the analysis: learning challenges, learning opportunities, and long-term impact. Students identified four categories of learning challenges: communication, visualization, clinical exposure, and heat. Five learning challenges identified by faculty were: faculty burnout, service delivery challenges, material wastage, teaching difficulties, and lack of comprehensive care. The five learning opportunities students identified were autonomy, preparedness, efficiency, safety, and personalized feedback. Learning opportunities identified by faculty were time management, autonomy, and preparedness. Three categories of long-term impact on students identified were future opportunities, adaptation, and postgraduation plans. Faculty identified apathy, career re-evaluation, and adaption as the long-term impact of COVID-19-related changes.

**Conclusion:**

Although the new COVID-19-related infection control procedures and regulations in the dental school clinical setting come with learning challenges, students and faculty also saw learning opportunities through increased autonomy, preparedness, and efficiency. The impact of COVID-19 extends beyond the current learning experiences as it may modify students' long-term plans.

## 1. Introduction

Coronavirus (COVID-19) is a respiratory virus that can be transmitted through aerosol, droplets, and contact routes, even in asymptomatic patients [1]. The risk of acquiring and transmitting the virus is high in dental settings due to aerosol production and close physical proximity to patients [2]. According to the World Health Organization, dental healthcare providers pose a high exposure risk to COVID-19 [3]. In March 2020, state health departments suspended all elective dental treatments to mitigate the spread of COVID-19. To control the spread of COVID-19, the American Dental Association, World Health Organization, and Center for Disease Control and Prevention published new infection control guidelines for oral healthcare providers during the COVID-19 pandemic [4–6]. These advanced infection control guidelines included physical distancing in waiting areas, COVID-19 screening, preprocedural mouth rinse, limited clinical care, and increased personal protective equipment (PPE).

Dental education, just like any other profession, has been impacted by the COVID-19 pandemic. Many dental schools have evolved curricula to overcome pandemic-related challenges. Streamed online lectures were used instead of sizeable in-person class gatherings. For smaller group presentations, interactive webinars and tutorials were used [7]. Clinical learning has been the most challenging aspect of dental education during the pandemic [7]. The vast majority of the students lacked the confidence to treat the patient without a hands-on clinical sessions [8].

The dental schools' most crucial issue is providing students with a safe clinical learning environment while eliminating the risk of spreading infection [9]. The mandatory guidelines while providing dental treatment included: (1) taking the patient temperature and symptoms of COVID-19 travel and contact history, (2) wearing PPE, including a surgical cap, surgical gown, N95 ventilator/surgical mask, and face shield, and (3) high power suction and rubber dam isolation for aerosol producing procedures [1, 4–6]. Dental students are divided into cohorts to minimize the risk of infection and close contact in indoor venues. Advanced PPE protocol, a reduced number of patients, and aerosol-producing procedures have been implemented to enable a clinical learning environment.

Dental clinical education is based on one-to-one interaction between faculty and students. Students must demonstrate diverse knowledge, skills, empathetic clinical behavior, and professionalism when treating patients [10, 11]. The clinical faculty supervises, monitors, guides, and provides feedback on clinical skills and behaviors [1, 10, 11]. PPE is a critical infection prevention and control measure for healthcare workers. However, these increased robust measures may hinder faculty's ability to explain and students' ability to understand complex concepts and technical competencies and show compassion and care [12].

However, these increased robust measures may hinder the faculty's ability to explain technical competencies. Students may have difficulty grasping complex concepts and also display empathy. The current study explored how COVID-19-related clinical changes affect student clinical learning experience from faculty and students' perspectives.

## 2. Methods

### 2.1. Study Design

A qualitative approach for inquiry was used in the study. A semistructured interview guide was used to collect qualitative data through focus groups.

### 2.2. Participants and Setting

Participants in this qualitative study were both students and faculty. Full-time clinical faculty and third- and fourth-year dental students with experience working within the clinic before and after COVID-19-related changes were implemented were invited to participate.

### 2.3. Sampling

The invitations to participate in the study were emailed to all third- and fourth-year students and full-time faculty. The invitation email included information about the purpose of the study, procedures, and ethical considerations. Participants were informed that the sessions would be conducted on ZOOM (ZOOM Video Communication, Inc., San Jose, CA, USA) and would be audio and video recorded. Participants were also informed that participation in the study was voluntary, anonymity would be protected by using pseudonyms, and all video and audio recordings would be deleted after transcription. Participants interested in participating in the study were given the dates and times to sign up for the online focus group sessions. Participants were informed that the session would last 60–90 min but could run a little longer. All participants completed an informed consent allowing the video and audio recordings of the focus group session. No incentive for participation was offered. All recruitments and data collection were completed in the summer of 2020; one semester after the implementation of COVID-19-related PPE changes.

### 2.4. Data Collection

A demographic survey was collected, which included age, gender, ethnicity, and current year of dental school. An identification number was assigned to each participant. Questions in the focus group revolved around dental students' and faculty's perceptions and experience with the COVID-19-related infection control procedural changes in the clinic setting and their impact on clinical learning.

A semistructured interview guide was used to reduce researcher bias (Table 1). All interviews were conducted by two interviewers (ME, SR). Before the start of the focus group, participants were given an overview of the study along with their rights and were informed that they could skip any questions that made them uncomfortable.

All the questions were open-ended and unambiguous; no leading questions were posed to ensure truthful responses. The researchers avoided misleading comments and distorting responses by staying neutral in their reactions to responses. Prompts were utilized depending on participants' responses to provide clarifications. After the question was asked, each participant was allowed to respond before moving on to the next question. At the end of the focus group session, participants were allowed to add anything related to their learning and teaching experiences. Sessions were conducted over ZOOM (ZOOM Video Communication, Inc., San Jose, CA, USA). The focus group sessions were recorded and *transcribed verbatim*. Two independent researchers conducted *verbatim transcription* by writing down every word, including pauses and expressions of emotions.

### 2.5. Data Management and Analysis

Data were analyzed and organized through stages. In the first stage, the team members verified the accuracy of the transcripts with the audio/video recordings. In the second stage, researchers used color coding to define similar patterns and themes connected to the participant' s responses. In the third stage, the thematic data analysis was identified; similar codes were grouped into thematic categories (words and short phrases) in the text; related categories were then combined into key themes [11]. Two independent team members who were not the interviewers transcribed, coded, and categorized the data. In the last stage, researchers used conference calls and electronic communication to discuss differences in coding, categories, and themes and reached a consensus.

## 3. Results

### 3.1. Participants

Twenty-two students (18.3% response rate) and 12 faculty members (40.0% response rate) were divided into six focus groups. Fourteen students were in their third year and eight were in their fourth year. Thirteen students identified as females and nine identified as males. All except one student were between 24 and 34 years old. All the faculty who participated in the study were full-time clinical faculty at the dental school. Five faculty members identified as females and seven as males. All faculty members were between the ages of 35 and 65 years. Participant' s characteristics are shown in Tables 2 and 3.

**Figure 1:**
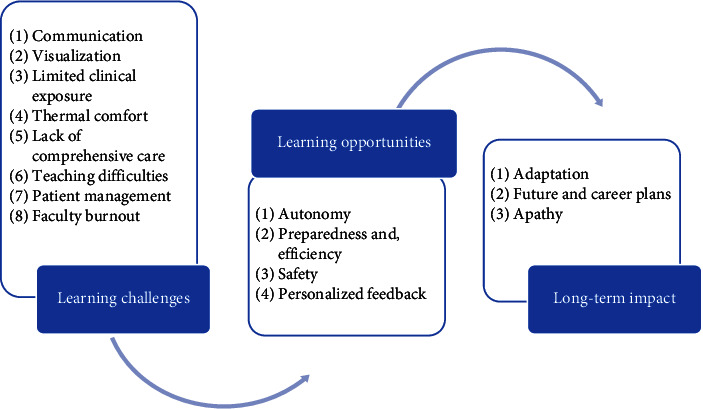
Thematic map from reflections.

Three major themes emerged from the data: learning challenges, learning opportunities, and long-term impact (Figure 1).

### 3.2. Learning Challenges

Students identified four learning challenge categories. Those categories were communication, visualization, clinical exposure, and heat. Four additional learning challenges identified by faculty include faculty burnout, material wastage, teaching difficulties, and lack of comprehensive care. Four learning challenges identified by faculty and students were communication, visualization, less clinical experience, and thermal comfort. Additional challenges identified only by faculty were lack of comprehensive care, teaching difficulties, patient management, and faculty burnout.

#### 3.2.1. Communication

Both students and faculty perceived that increased PPE negatively impacted verbal communication between students, faculty, and patients. Participants reported having difficulties hearing each other.

One student said *“I would also like to add that I struggled to hear people.” “That honestly might be the biggest one for me, especially working with faculty members who don't talk very loud.”*

Participants felt that they had to repeat themselves numerous times and needed clarification on whether the other person understood the instruction. Students reported that listening to faculty was hard and that most clinical instructions were assumed rather than being listened to. Older faculty reported needing help hearing and speaking loudly and avoiding communicating and giving instructions unless imperative. Participants reported facing challenges in understanding people who spoke softly, fast, or with different accents. Participants reported listening fatigue due to the increased effort needed to listen.

*“I have something to say, and I' m sure you're well aware that I wear hearing aids. I'm sure there are a lot of people who have good hearing. There's something called ‘listening fatigue' where you're just trying so hard to listen all day long with … and you're trying to read facial expressions just at the eyes. When you're looking intensely and focusing so hard, at the end of the day it's taxing on your energy.”*

In addition to verbal communication, the added layers of face mask, shield, and eyewear have taken away the nonverbal communication abilities between providers. Faculty reported that they rely on nonverbal cues to assess if students need additional instruction during procedures. Faculty believe that since they cannot see students' facial expressions, it is challenging to gauge students' understanding. They cannot assess if the student is concerned, scared, or needs more independence or assistance. Pediatric faculty stated that nonverbal communication through facial expression was a primary source of communication between providers and children. New PPE protocols have impeded this form of communication, impacting pediatric patient compliance. One faculty said:

*“Sometimes just by looking at a student face whether they are getting it, concerned, scared. I use that information especially if we are doing something critical in oral surgery. Oftentimes, I use that information to direct how I am teaching and that is lost. That is definitely lost.”*

Another faculty said:

*“we rely a lot on facial expression to converse with patients to understand how they are doing, and to convey information, so that's been a lot more difficult with the PPE.”*

#### 3.2.2. Visualization

Participants reported having difficulties with visualization. The inability to put the light inside the loupes fogging of loupes and a layer of the shield in front of loupes created glare and skewed view causing difficulty in visualization. Students reported struggling to defog their glasses to work on a patient properly. Faculty said that students' loupes, along with the face shield, were sometimes so fogged up that even patients were concerned if the provider could actually see what they were doing.

*“I saw several occasions where it was ridiculously foggy and the patients themselves said kind of like, ‘Why … You can't … How can you actually see what you're doing?'”*

*“But just visibly not being able to see as well, and then having the shield on top of that, the reflection from the lights sometimes could make your field of view a little bit skewed.”*

#### 3.2.3. Less Clinical Experience

Students and faculty reported that their time for essential clinical experiences had been reduced. More extended patient check-in protocols and time spent reviewing the safety protocol questionnaires' have decreased procedures time.

Students feel rushed due to time restrictions, multiple checks, and wait times for faculty, needing more time to leave the operatory to get materials and not getting quick consults from specialists. This has affected the efficiency of treating the patients. Students felt anxious and felt that they might have to do a residency to gain more clinical experience.

*“We just don't treat as many patients as we use because of the logistics of every part of Gowning… I think for some other people, this is a really, really nerve-wracking time. They're feeling like they want more experience and more time in a GPR. But because so many people applied and that they' re maybe not getting in, I think there's definitely … It's going to be an interesting transition period for some people.”*

#### 3.2.4. Thermal Comfort

Students and faculty reported feeling hot with an added layer of PPE, double masking, and face shields, making them very uncomfortable during clinic sessions. The body's inability to get rid of sweat in PPE increases the body temperatures causing fogging of the face shield and loupes and making it very difficult to work.

*“But in terms of PPE, I would say, for me, my biggest issue starting off at least in the summer/early fall … and actually, all the way into fall, was how hot I was while I was working. That was a real issue for me because I was one of the people that tested the new protocol, and especially when we were doing double masks for the very short time that we were, I had a really hard time with that.”*

#### 3.2.5. Comprehensive Care

Faculty believes comprehensive care is being neglected, treatments are deferred, and follow-ups need to be done on time. Patients are getting different care providers every appointment due to limited time clinical time slots for students.

*“Every single patient is different. Every single tooth is different. Every interaction is going to be different. If you start having very limited interactions, your result is a lesser education. You graduate from this university, or any university, with less skill to treat your patients in the real world.”*

#### 3.2.6. Teaching Difficulties

The faculty believes that due to the risk of cross-contamination and changes in the PPE between patients, they need to be more flexible in providing many hands-on instructions. To avoid changing PPE between patients and spending time in the doffing area, faculty give more verbal rather than show-and-tell hands-on instruction. Faculty believe that specialties like pediatric and oral surgery, which require teaching with a lot of hands-on instruction, are negatively affected by COVID-19-related changes.

*“It's affected our flexibility. We can't be flexible in helping the students as much as we used to, or flexible in the treatment we give the patients because if we're not on that non-aerosol side, or they're in aerosol, then they have to come back again and it's just … It's made it more inconvenient for everybody.”*

Participants believe that eliminating pre- and post-clinic huddles affects student learning by taking an important learning tool. Students believe huddles provided an excellent opportunity to ask questions about patients or procedures, and debriefing after patients provided a chance to discuss different technique materials. All these learning opportunities are impeded. The faculty believes that it was beneficial for the students to talk about the procedures right after patient dismissal when it was fresh in everyone's mind. Most of the discussion happens a few days later on Zoom, and they forget the minute details by then. Conversely, some students did not perceive the lack of immediate feedback as a significant barrier to their learning.

*“I would say not having the end of the day huddle has had an effect on the learning process. Because, I have definitely learned from other students, their triumphs, failures, are usually expressed at the end of the day.”*

#### 3.2.7. Patient Management

Faculty felt that students were forced to focus more on procedures than holistic patients-centered care. Faculty believed that students need more training in personal connection, chairside manners, and skills to build rapport, effective communication, and empathy, which are essential components of patient adherence in private practice

*“…We were trained with empathy on our sleeves and we're very verbal, and hands-on exactly, and we had facial expressions. We could sympathize with the patient. We could say things with our eyes and our facial expressions, and these students now are going to be trained to be behind a screen basically, like The Wizard of Oz… They're not going to know how to do all the things that they really should be doing to be a really effective provider.”*

#### 3.2.8. Faculty Burnout

Faculty reported feeling overworked and exhausted with the additional layers of PPE. Faculty reported long clinic hours and added layers of PPE, making it challenging to see and hear daily. The faculty shortage has affected their tempers, and they feel burned out.

*“Oh, I'm still burned out. I'm still dealing with that, and we just got back from vacation. But I'm going to be … I'm on aerosol every day of the week. I'm in PPE with two masks every day of the week. No one is getting a break still.”*

### 3.3. Learning Opportunities

Four categories of learning opportunities were identified as an outcome of the interviews. Those were autonomy, preparedness and efficiency, safety, and personalized feedback.

#### 3.3.1. Autonomy

Students believed they got more autonomy, given that faculty had to change PPE between patients and could not spend much time with each student. Students felt faculty provided them more freedom by doing fewer step checks and more verbal than hands-on instructions, which helped them be more independent.

*“There's a little bit more autonomy because of the logistics that are involved. But I think that's probably also on a case-by-case basis.”*

#### 3.3.2. Preparedness and Efficiency

Students felt that the COVID-19 restrictions made push them to be more prepared. They believe that since they were not allowed to leave their operatory once seated, they were better prepared and did their due diligence beforehand. Students reported keeping better track of the schedule as well as material procurement. They made sure that faculty was only called over when they were undoubtedly ready.

*“So, it's definitely a good daily exercise, getting exactly what you need, planning for your contingencies and planning for it all in the operatory.”*

*“It just kind of pushed me to make sure that when a faculty comes over it is just exactly what they want to see so they don't have to deglove and demask. I guess that is a blessing in disguise maybe in the long run, but it definitely took a learning curve.”*

Being pushed to be prepared in addition to other changes like a single system to call faculty over for check, specialists present on the clinic floor, being prepared with all the material handy, and time-check put in by faculty had made the clinic sessions run very efficiently.

*“But one thing that I have found that I think has made the clinic run a little bit more efficiently… I think the hand signals, like the two fingers up for a doc and three for a hygienist, has made things run a little bit smoother… It's annoying that we can't leave our operatories, but I think that's something that would be worth implementing past the COVID-19 time.”*

#### 3.3.3. Safety

Students and faculty felt very comfortable treating the patients because of the additional layer of PPE made them. The faculty thought that patients felt safer visiting the clinic when they saw the extra layer of protective PPE in place.

*“I'd say overall though, feeling safe and your patients feeling more safe by seeing all of the PPE was, you know, the greater good I suppose.”*

#### 3.3.4. Personalized Feedback

Students believe that faculty provides more personalized feedback than group discussions. Students believe that faculty are providing them with in-depth personal one-to-one constructive feedback, which has helped them with confidence and made the interactions a positive learning experience. Instead of getting in that big huddle, if the faculty and students can come together after the appointment, they go a little more in-depth than they would in post-op huddles.

*“Talking with the faculty that have time and experience immediately after and they get on a much more personal level than they would rather in the group.“*

### 3.4. Long-Term Impact

Students and faculty identified three categories of long-term impact of the COVID-19-related changes. These include adaptation, future and career re-evaluation, and apathy.

#### 3.4.1. Adaptation

Students and faculty believe that there were many changes in delivering dental care and adjusting to the new protocol, but everyone has adapted. The faculty thought that COVID-19 made students realize there would be dynamic changes and circumstances in their practices throughout their lives. It is a good experience for them to know how to adapt and modify to change. Students believe that changes in circumstances have made them strive to be better dentists in the future.

*“Despite all the cons that have happened that have made a change in our curriculum, it has made us better dentists and that is something no one will admit. Being able to adjust our dental education because of these standards is not something most other classes of dentist in the past have ever dealt with, so that is something we should also keep in mind.”*

#### 3.4.2. Future and Career Plans

Students felt apprehended and started questioning their career choices and adjusting their plans. Many students plan to apply for advanced residency programs. Students were also worried about the job market and finding a job that would pay them enough to cover their debt. Faculty reports that many students regularly discuss the uncertainty of the future.

*“I think that's what a lot of students are struggling with. We have had conversations because they're like, ‘If this is going to be my life, do I really want to do it anymore.'”*

*“I feel a lot more students will have the proclivity to go for the GPR for extra practice and boost their confidence.”*

Students believe that many older dentists contemplating the idea of retiring are retiring now. Students and faculty feel the pandemic may allow students to own private practices.

*“I don't think it has necessarily outright changed my plans directly, but I am definitely more cautious of the fact of practice ownership with all the in-carried cost that can happen.”*

#### 3.4.3. Apathy

Faculty members believe that apathy is pervasive among dental students, evidenced by decreased empathy. They believe that added protocols have affected student communication, patient–doctor relation, courtesy, energy, friendliness, and chairside manners. Students are treating patients as an entity rather than a person.

*“I think it affects us greatly! We are not a person as much as we are an entity!”*

*“That conservation between the student and them that used to happen about their dogs or grandchildren still takes place but doesn't happen as much. Not that it's an intrical part of dental education but it's that connection you form with the patient that allows them to trust you!”*

## 4. Discussion

This study aimed to explore how clinical changes related to COVID-19 may affect the student learning experience. COVID-19 modifications made in the clinic brought about a lot of challenges, with difficulty in communication and time restraints for patient treatment. However, it allowed students to get more personalized feedback, be more prepared, and have autonomy. The study also highlighted that the pandemic would have a long-term impact on students' adaptability, empathy, and future goals.

The dental profession has modified its standard PPE protocols to reduce the risk of COVID-19 spread. The rigorous preventive measures combined with the increased risk of infection have affected all aspects of dentistry, especially dental education [12]. Our study indicated that students faced many challenges while providing patient care with added layers of PPE implemented because of COVID-19. Clinical supervision, described as the “provison of monitoring guidance and feedback on personal, professional and educational development in the context of patient care,” [11, 13] is mostly affected because of difficulty in communication among faculty, students, and patients [10]. Faculty and students both believe their interaction with each other was minimal, making it difficult to learn on the clinic floor effectively. Faculty and students were both negatively affected by the shift in teaching modalities [14]. Faculty usually rely on nonverbal clues from students to assess when the interjection is needed or required while providing patient care. New protocols have made it impossible to use nonverbal clues, and faculty and students perceive that it has negatively impacted their learning and teaching experience. In addition to student–faculty communication difficulties, communications with patients were also affected. From the initial consultation onwards, communication between patient and provider is key to an excellent clinician–patient relationship [11, 15]. Students perceived that face shields and masks made it challenging to discuss treatment options or effectively deliver treatment plans.

Faculty believe that by following the new guidelines, they cannot perform any clinical demonstration, which has impacted students' acquisition of fine motor skills and critical thinking. Farrokhi et al. [16] also reported the negative effect of decreased clinical time in providing patient care on students' clinical skills. The standard view shared by students and faculty was that reduced times with patient care, lack of hands-on instruction, and pre- and post-operative discussion had affected the patient-centeredness and comprehensive care experiences. Loch et al. [17] and Farrokhi et al. [16] in their studies, reported that faculty believe that they spend more time worrying about safety related to COVID-19 than actual teaching. Conversely, some participants thought that the lack of direct faculty supervision gave them more autonomy. They felt self-motivated to make self-directed decisions and be prepared to take responsibility for their patients and self-learning. Francesca et al. [18] also reported a positive impact on students with a shift toward productive learning strategies and increased engagement.

Students felt they needed to acquire more clinical skills because of the restrictions and felt less confident starting their professional careers [19]. Rodriguez-Vamvas et al. [20] reported distress and lack of clinical preparedness among graduating students. Hattar et al. [21] reported that two-thirds of the students preferred to be supervised following graduation. Direct patient care impacts students' clinical skills and affects their interprofessional interactions. Unsurprisingly, students reported uncertainty and a lack of preparedness for the future. Increased stress levels and uncertainty of the future during COVID-19 have been reported by other studies as well [15, 17]. Faculty in the present research reiterated the same thoughts and to overcome the clinical weakness and build self-confidence encouraged students to seek advanced training, additional courses, and workshops.

Empathy is critical in establishing a relationship between healthcare providers and patients. A wealth of studies have found a direct relationship between empathy, patient satisfaction, patient self-efficacy, treatment acceptance, and compliance [22–26]. A decrease in dental anxiety and fear has also been reported in studies where dentists showed some empathy toward patients [27, 28]. Factors contributing to the decrease in empathy levels among dental students are reported to be burnout, increased work-to-study load, and time constraints in providing patient care. Faculty in the current study perceived that increased stress associated with COVID-19 protocols, time restraints in completing the procedure, difficulty in communication, and lack of interpersonal relations have changed current students' empathy levels. Even though the American Dental Education Association has stated empathy as the second clinical competency for dental training, faculty is concerned that students are unaware that empathetic behavior can impact their patient communication, clinical practice, and patient retention. To increase the level of empathy among students, faculty believes that they should not just focus on clinical skills but also provide feedback on communication skills.

Dental students have one of the highest debt-to-income ratios among healthcare professionals. Unemployment has been at the highest level since the depression era, and COVID-19 has significantly affected dental practice values [29]. Students are apprehensive about meeting their financial obligations [30]. There is uncertainty about patients returning for regular care and the possibility of elective procedure reduction with increased extraction and a low-cost prosthesis. Both can have a massive negative financial impact on many practices. A level of uncertainty and economic implications to remodeling the practices to meet the new guideline has encouraged small business owners to retire early [31]. Although students may face looming debt and unemployment, they did hint at a silver lining where they were allowed to own their private practices early in their careers.

Even though COVID-19 has brought a lot of challenges, it also brought some silver linings and opportunities. A wealth of studies suggested the need for innovative teaching from online learning to hybrid approaches, simulation critical reflection, and teledentistry [32–36]. The current use of teledentistry has been limited to improving access to and establishing homes for underserved children [36]. With the involvement and advice of public health officials, insurance companies are now providing options for reimbursement for teledentistry. This brings an opportunity to utilize teledentistry for patient screening, providing consultations, diagnosing, presenting treatment plans, and identifying dental emergencies [37, 38]. The minimally invasive dentistry concept is becoming relevant in the COVID-19 era. Studies have indicated that COVID-19 has allowed advocacy for conservative and less invasive procedures, especially among high-risk populations [38, 39]. Faculty and students in the current study also indicated the need for teledentistry and systems with minimal aerosol production.

Two main limitations of the study are its qualitative design and the fact that participants were from one school. Other schools may have different clinical changes that may affect the clinical operations differently, and this will limit the generalization of the findings. Since the pandemic is still evolving, future studies can investigate the long-term impact of the new guidelines on clinical education and student career paths.

## 5. Conclusion

In conclusion, the new COVID-19-related infection control procedures brought immediate learning challenges, which may have a long-term impact on dental education but also brought up some opportunities. The impact extends beyond the current learning experiences into the future career plans of dental students.

Future studies exploring COVID-19-related infection control procedures and their long-term impact on students' clinical skills are warranted. The current study highlights the need to focus on prevention, patient empowerment, and increasing the role of teledentistry is recommended for improving oral health outcomes.

## Figures and Tables

**Table 1:** Interview guide.

(1) How has the additional PPE due to COVID-19 impacted your overall clinical learning experience? (2) How has the additional PPE due to COVID-19 has affected your learning experience on the clinical floor? *Communication* How has the additional PPE due to COVID-19 has affected your interaction with faculty on the clinic floor while treating the patients? (1) What about nonverbal communication? (2) What about paraverbal communication (tone of voice, clarity, attentiveness, volume, pacing of questions, and voice inflection)? *Clinical instructions* How has the additional PPE affect the clinical instruction you are receiving? (1) Preprocedure. (2) During-procedure: clinical demonstrations by the faculty. (3) Postprocedure. *Discussion and feedback* How has the additional PPE due to COVID-19 has affected your posttreatment daily feedback (huddle, after treatment discussion)? *Patient interaction* (1) How has the additional PPE due to COVID-19 has impacted your interaction with patients? (2) Delivery of the treatment plan. (3) Preprocedure discussion. (4) Postprocedure instruction. How has the impact of COVID-19 on your clinical learning experience affected your future plans? Is there anything else you would like to add?

Interview guide questions used during the focus groups.

**Table 2:** Student participants' demographics.

Demographic characteristics	*N*	Percentage
Year		
D3	14	67
D4	7	33
Age		
25–34	20	95
35–44	1	5
Gender		
Male	9	43
Female	12	57
Ethnicity		
Caucasian/White	12	57
African American/Black	1	5
Hispanic/Latino	1	5
Asian	7	33

**Table 3:** Faculty participants' demographics.

Demographic characteristics	*N*	Percentage
Age		
35–44	3	25
45–54	2	16.6
55–64	3	25.0
Over 65	4	33.33
Gender		
Male	7	58.33
Female	5	41.6
Ethnicity		
Caucasian/White	10	83.3
Asian	2	16.6

## Data Availability

Data used to support the findings of this study are available from the corresponding author upon request.
